# Surgical management of a type A aortic dissection in a pregnant patient

**DOI:** 10.1002/ccr3.7943

**Published:** 2023-09-22

**Authors:** Julian A. Gordon, Michael C. Larkins, Melisa Pasli, Sunny R. Cai, Adam C. Celio, Michael J. Bates

**Affiliations:** ^1^ Brody School of Medicine at East Carolina University Greenville North Carolina USA; ^2^ East Carolina Anesthesia Associates Greenville North Carolina USA; ^3^ Department of Cardiovascular Sciences East Carolina University Health Greenville North Carolina USA

**Keywords:** anesthesia, cardiothoracic surgery, cardiovascular disorders, obstetrics/gynecology

## Abstract

Despite emphasis for emergent surgical treatment of Stanford type A aortic dissections, pregnant patients that are clinically stable may safely receive a staged approach instead, with delivery followed by delayed dissection repair.

## Graphical abstract

An initial CT demonstrating a Stanford type A aortic dissection in a pregnant patient.

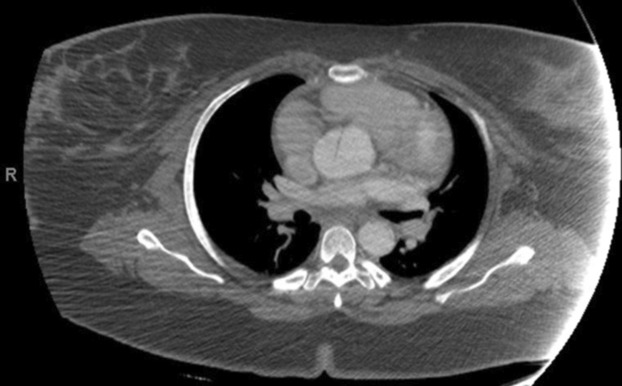

## 1 INTRODUCTION

Aortic dissection (AD) is a rare but life‐threatening event in which a tear in the intima of the aorta allows circulating blood to enter between the layers of the vessel.^1^ Stanford type A ADs involve the aorta proximal to the origin of the left subclavian artery and classically present with pathognomonic “tearing” chest pain, though some cases may present more insidiously.^2^ These dissections are associated with a 1%–2% increased mortality rate per hour, leaving fastidious diagnosis through either computed tomography angiography (CTA) or echocardiography a critical component to survival.^1^, ^3^ Treatment consists of pain control, beta blockade for heart rate, blood pressure management, and typically emergent surgery.^1^

While hypertension and connective tissue disorders are well‐recognized risk factors for AD, other risk factors, such as pregnancy, may not be thoroughly understood. Nonetheless, gravidity remains a critical consideration, as approximately 50% of the dissections seen in women under 40 years of age actually occur during pregnancy.^1^, ^4^ Furthermore, the most common time periods of the peripregnancy period are the third trimester and postpartum period.^5^ Both diagnosis and management of AD vary in the pregnant population as well, with altered maternal physiology and concern for fetal well‐being necessitating special consideration from clinicians. Although an abundance of literature exists pertaining to ADs in the nonpregnant population, data are inadequate regarding how this life‐threatening condition should be clinically approached in pregnant patients. Pregnancy‐related ADs have been reported as representing 0.3% of all ADs and 1% of ADs in women overall.^5^ Furthermore, AD in pregnancy is reported as occurring in only 0.0004% of pregnancies.^6^ The majority of these ADs were found to be the result of an aortopathy not diagnosed prior to pregnancy. We sought to present a rare case of an AD in a third‐trimester pregnant patient.

## 2 CASE DESCRIPTION

**FIGURE 1:**
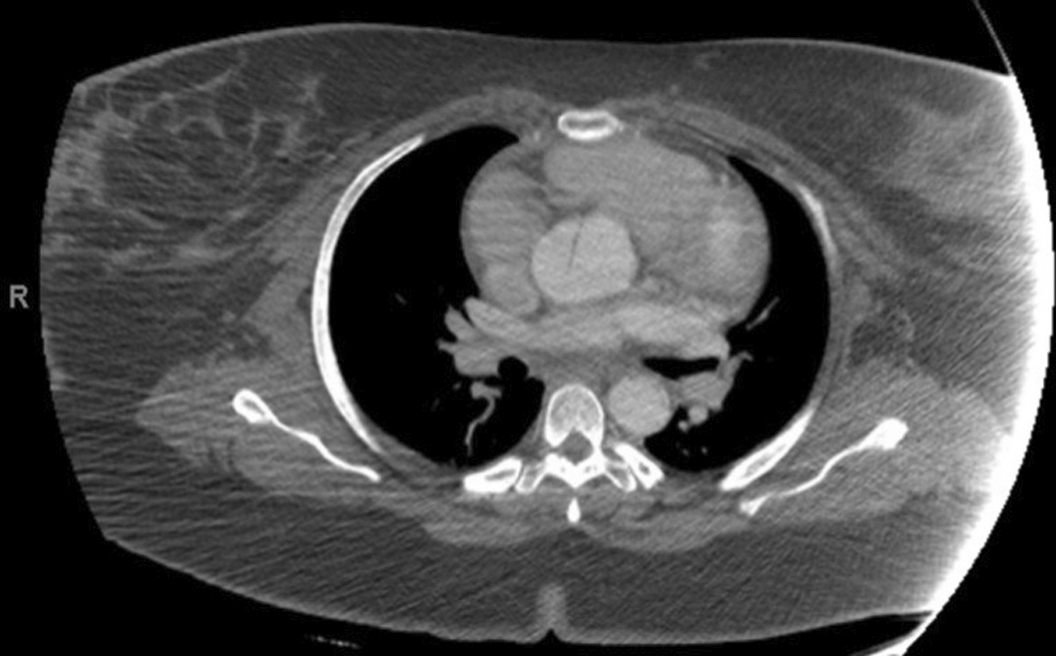
Initial computed tomography angiogram of the patient's chest obtained on hospital admission Day 3, showing a Stanford type A aortic dissection involving the ascending aorta.

**FIGURE 2:**
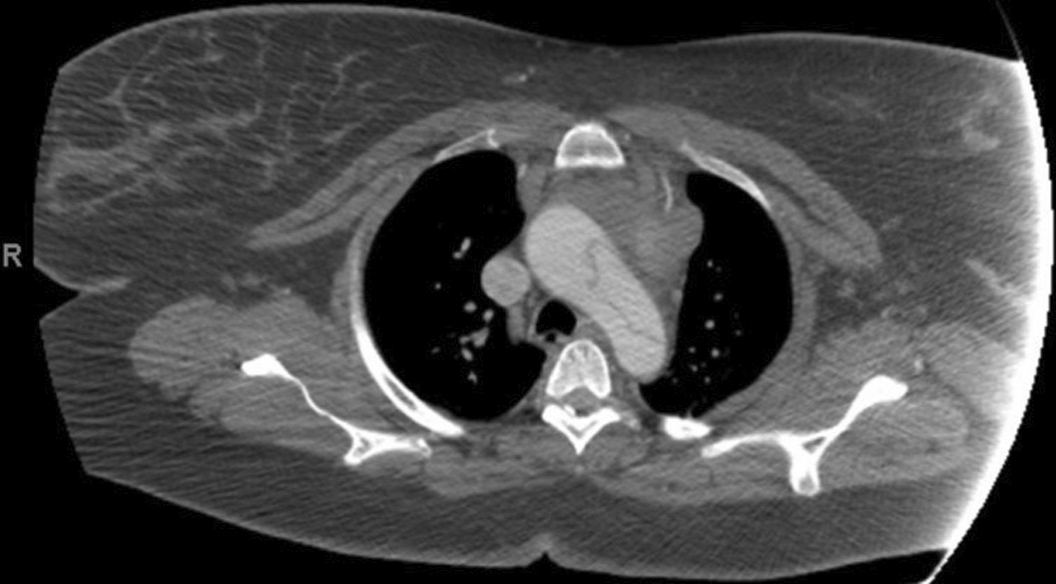
Initial computed tomography angiogram of the patient's chest obtained on hospital admission Day 3, showing a Stanford type A aortic dissection involving the aortic arch.

A 37‐year‐old G8P5 African American female at 31 weeks' gestation and with a past medical history significant for hypertension, gastric bypass surgery, gastroesophageal reflux disease, and gestational diabetes mellitus presented emergently to an outside hospital with abdominal pain radiating to the neck with right arm numbness and weakness, as well as diarrhea, nausea, and emesis. Workup revealed an unremarkable electrocardiogram, normal troponins, and unremarkable chest x‐ray. Hypotension 70/46 mm Hg was noted, and the patient was admitted with a diagnosis of gastroenteritis with concern for sepsis; remaining vitals at admission were as follows: respirations 22/min, pulse 63 beats per min, temperature 98.4 °F, and SpO_2_ 99% on room air. She was initially kept under observation and treated with fluids and antiemetics. Two days later, in conjunction with persisting abdominal pain, the patient was found to have significantly lower blood pressures in the right upper extremity (65/48 mm Hg) compared to the left upper extremity (122/58 mm Hg). CT Angiogram revealed a Stanford type A AD involving the ascending aorta, aortic arch, descending thoracic aorta, and abdominal aorta with a dissection flap just below the origin of the superior mesenteric artery (Figures 1 and 2).

**FIGURE 3:**
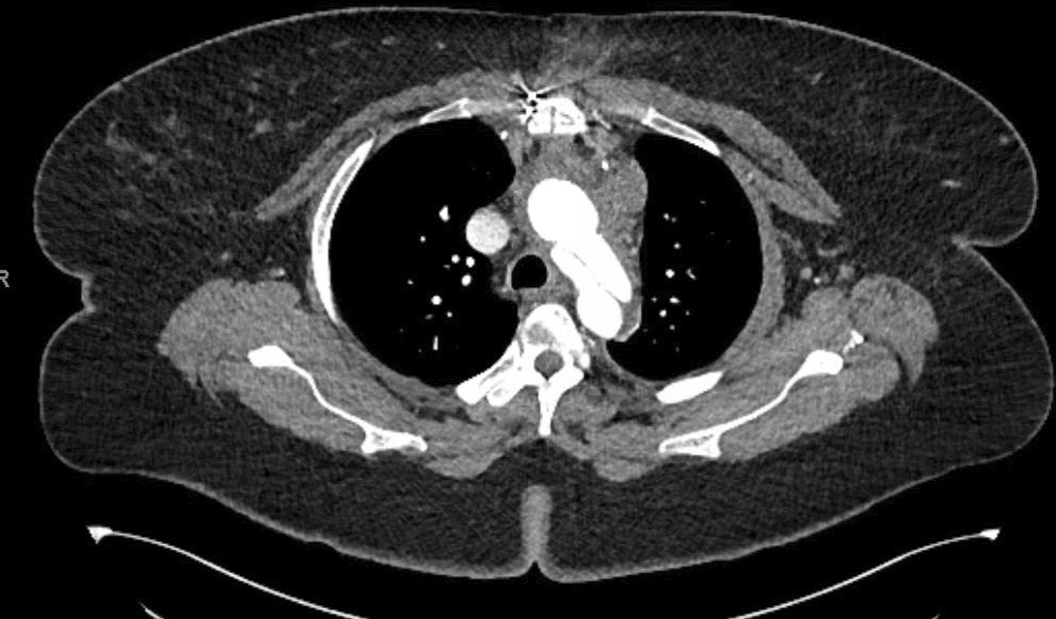
Postoperative computed tomography angiogram of the patient's repaired ascending aorta and proximal hemiarch, obtained 1 month after surgical repair of type A aortic dissection. The remaining, unrepaired flap distal to the repair can still be seen, also present in the descending and abdominal aorta.

The patient was transferred to our institution where a multidisciplinary team of obstetricians, cardiac surgeons, and anesthesiologists decided to use a staged approach due to her clinical stability. Vitals at admission to our institution were as follows: blood pressure 131/57 mm Hg, pulse 67 beats per minute, respirations 22/min, temperature 98.5 °F, and SpO_2_ 99% on room air. The patient underwent an emergent cesarean section under general anesthesia. Concurrently, transesophageal echocardiography (TEE) was performed and showed preserved left ventricular systolic function (ejection fraction 60%), mild aortic regurgitation, and moderate mitral and tricuspid regurgitation. Importantly, the TEE indicated no pericardial effusion. The newborn was healthy and transferred to the NICU as a precaution. The patient was extubated and recovered from the cesarean delivery and 3 days later, the patient underwent a hemiarch replacement to repair the type A dissection, accomplished using 26 mm Gelweave tube graft. This repair was limited to the ascending and proximal aortic hemiarch; a CTA of the repair can be seen in Figure 3. The patient was extubated the following day, soon weaned from vasoactive medications and cardiopulmonary bypass, and had an uneventful recovery. She was discharged to our institution's inpatient rehabilitation service on post‐operation day 12; vitals at discharge were noted as blood pressure 129/75 mm Hg, pulse 90 beats/min, temperature 98.8 °F, respirations 22/min, SpO_2_ 98% on room air. Retention sutures were left in place after the dissection repair and subsequently removed on an outpatient basis with no complications reported. Written informed consent was obtained from the patient for their anonymized information to be published in this article. A timeline of our patient's admission, transfer, and subsequent hospital course can be found in Table 1.

**TABLE 1:** Timeline of our patient's treatment course, starting with presentation to outside institution and ending with discharged from inpatient rehabilitation service at our institution.

Day	Event
1	Patient presents to outside hospital ED complaining of chest/abdominal pain, with hypotension as low as 71/37 mm Hg noted. Cardiac workup is unremarkable. Patient is ultimately admitted for presumptive gastroenteritis with concern for sepsis.
3	Patient continues to have intermittent hypotension despite administration of fluids. A difference in right and left lower extremity blood pressure of about 60 mm Hg systolic is noted. Subsequent CTA revealed Stanford type A aortic dissection (AD). Patient is transferred to the authors' institution for further evaluation and a higher level of care. After transfer decision is made for staged approach for surgical management of patient, and patient undergoes successful emergent cesarean section. Patient then sent to cardiovascular intensive care unit (CVICU), given maintenance fluids and 1 unit of packed red blood cells, and transitioned from esmolol to labetalol drip. Point of care transesophageal echocardiogram (TEE) reveals dissection in the ascending aorta traveling distally to the abdominal aorta, with a moderate pericardial effusion and ejection fraction (EF) of 60% noted.
4	Patient's labetalol dose is maxed out and cleviprex and esmolol eventually added to control patient's heart rate and blood pressure.
6	Patient taken for aortic dissection surgical repair. Surgery is successful with no complications noted; preoperative TEE unchanged from previous TEE.
12	Patient is discharged to our institution's inpatient rehabilitation service for postoperative recovery. Patient is kept on diuretics and antihypertensives with a systolic blood pressure goal of <130 mm Hg.
14	Patient is discharged from inpatient rehabilitation to home after uneventful 2‐day stay.
26	Patient follows up with cardiothoracic surgery service on an outpatient basis. Patient's surgical sites are well healed and repeat CTA shows aortic repair is stable. Patient's sutures are removed. Patient is given 12‐week postoperative lifting restrictions and a 12 month follow‐up.

## 3 DISCUSSION

Classically, ADs present with widened pulse pressure, unequal blood pressures in upper extremities, and pathognomonic “tearing” chest pain radiating to the back.^1^ However, recent literature has suggested these easily recognizable findings may not be so common, with one study finding only 50.6% of dissections to present with pain described as “tearing or ripping” and just 28.3% with radiating pain.^7^ Conversely, some cases present with vague symptoms, such as dyspnea and vomiting, which may easily be confounded with general malaises of pregnancy, as was the case with our patient initially.^7^, ^8^, ^9^ Individualizing risk factors in ambiguous settings such as these may make diagnoses more definitive. Our patient not only had chronic hypertension—a common risk factor for AD—but was also pregnant, which increased the likelihood of dissection from 1.24 per million in nonpregnant women to 14.5 per million in pregnant women due to the hyperdynamic state and hormonal effects on vasculature associated with pregnancy.^4^, ^8^

To expand on the pathophysiology of ADs in pregnant patients, increased sympathetic activity and activation of the renin‐angiotensin‐aldosterone system contribute to increased cardiac output and overall blood volume; this in turn puts more stress on blood vessels, especially the aorta, increasing susceptibility for AD during pregnancy and in the postpartum period.^5^ These changes peak during the third trimester and can exacerbate preexisting vessel wall defects, which can themselves be the result of an underlying, often not previously diagnosed condition such as Marfan syndrome or vascular Ehlers–Danlos syndrome. Furthermore, it is possible the increased concentrations of hormones related to pregnancy and the postpartum period, such as oxytocin, can influence the behavior of the blood vessel wall and therefore contribute to increased aortic dilatation and vessel defect formation.^10^

One of the challenges in diagnosing AD during pregnancy is imaging. While the gold standard for AD diagnosis has traditionally been CTA, both radiographs and CT scans are discouraged in pregnancy due to the potentially harmful effects of radiation to the fetus.^11^ In addition, contrast dyes utilized for angiography have been well associated with nephropathy, potentially exacerbating the already‐comprised state of the kidneys due to physiological changes of pregnancy.^12^, ^13^ Although the dose of radiation delivered by CTA is below the limit for fetal harm, thus leaving the American College of Obstetrics and Gynecology in favor of utilizing CT imaging in pregnant patients that require imaging.^14^ Other imaging alternatives, such as magnetic resonance imaging, can be utilized in pregnant patients with varying diagnostic accuracy and availability.^11^

The majority of pregnancy‐related AD are Stanford type A (57%–80%), necessitating surgical management; type B can be managed medically but may still require endovascular or surgical repair.^5^ Surgical planning for AD in pregnant patients is challenging, necessitating effective communication between the patient, anesthesiologists, obstetricians, and cardiac surgeons. Factors that impact clinical decision‐making include the onset and presentation of AD symptoms, hemodynamic stability, and fetal viability.^15^ Additionally, surgical repair is generally poorly tolerated by the fetus in utero, with fetal loss upward of 30% due to a complex response to stress and cardiopulmonary bypass.^16^ Although there are no official guidelines regarding the management of AD in pregnancy, some have proposed tailoring treatment based on gestational age, with concomitant delivery if the fetus is mature and gestational age is between 28 and 32 weeks.^4^ In contrast, the European Society of Cardiology recommends delivery starting at 26 weeks gestation.^17^ Additional literature has developed this strategy further by suggesting emergent cesarean section with sternotomy standby followed by surgical repair of the aorta within a few days.^18^

Staged repair of type A ADs in pregnant patients has been reported in the past, patients with gestational ages ranging from 24 to 36 weeks and the interval between delivery and repair of the aortic defect occurring in the context of patient stability.^19^, ^20^ In our patient's case, the clinical team decided to have the patient undergo cesarean section first, in case of any hemodynamic instability that may have developed during the procedure. This was decided in the context of the mother in this case report being relatively stable, the fetus having reached relative gestational maturity, and in keeping with the current literature. The added benefit to this staged approach is to allow for additional time for affected vessels to potentially heal or stabilize, if the mother in such instances can tolerate the delay in surgical repair.^20^ Had the mother been clinically unstable, the decision to perform emergent surgical repair with cardiopulmonary bypass would have been weighed against the inherent risk of fetal demise. Surgical repair following delivery was performed within 3 days of delivery, as the mother was clinically stable. Had the mother been clinically unstable, this interval may have been shorter or temporizing techniques, such as intra‐aortic balloon placement for counterpulsation, would have been considered. The utilization of such techniques would be limited as the patient's dissection was extensive, continuing down into the abdominal aorta. Additionally, because of the length of involved aorta and potential risk of attempting to repair the entirety of the defect, the surgery undertaken was a repair limited to the ascending aorta and proximal hemiarch.

Our study is a limited analysis of the optimal management of pregnancy‐related AD, in that we present a singular case. This adds to the literature regarding AD but is not a substitute for more rigorous analysis. Additionally, there was extended discussion on the risk–benefit of surgery with the fetus still in utero, as the patient was at 31 weeks' gestation and considered viable with cesarean delivery. Future studies should systematically investigate the efficacy and outcomes (both fetal and maternal) for both staged delivery and AD repair, and risk benefit of screening and/or prophylactic repair of patients with aortopathies/predisposing conditions for dissection. Many women with an aortopathy condition are not diagnosed until pregnancy or the postpartum period, in part due to many such conditions not having associated readily apparent physical characteristics, and in part also due to the inconsistency of patients with a pregnancy‐related AD having definitive genetic testing that shows risk for developing an AD.^5^ There is an obvious need to weigh the risks and benefits for increased surveillance and prophylactic treatment, which could be medical or surgical management. Given the rarity of pregnancy‐related AD, the added morbidity, mortality, and medical costs associated with such changes would need to be considered.

## 4 CONCLUSION

We present a case of successful surgical treatment of an AD in a pregnant patient after an initial delay in diagnosis treated with a staged approach of delivery followed by delayed dissection repair. ADs represent a potentially catastrophic complication of pregnancy. Although ADs are thought to have the hallmark easily identifiable tearing chest pain, atypical presentations may delay accurate diagnosis and clinical management. Increased clinical suspicion for AD is warranted in patients with corresponding risk factors, especially pregnancy. Treatment aims to mitigate the risk of both fetal and maternal demise, and typically includes emergent cesarean section and AD defect repair, though consensus regarding the timing of repair is currently lacking. While AD in the general population is well studied, future studies should aim to better describe how AD may present during pregnancy and how management differs in the gravid patient from that of nonpregnant patients.

## AUTHOR CONTRIBUTIONS

**Julian A. Gordon:** Conceptualization; formal analysis; investigation; writing – original draft; writing – review and editing. **Michael C. Larkins:** Formal analysis; writing – original draft; writing – review and editing. **Melisa Pasli:** Formal analysis; writing – original draft; writing – review and editing. **Sunny R. Cai:** Conceptualization; formal analysis; project administration; resources; supervision; writing – original draft. **Adam C. Celio:** Formal analysis; project administration; writing – original draft. **Michael J. Bates:** Formal analysis; project administration; supervision; writing – original draft.

## FUNDING INFORMATION

This research received no specific grant from any funding agency in the public, commercial, or not‐for‐profit sectors.

## CONFLICT OF INTEREST STATEMENT

The authors declare that there is no conflict of interest.

## CONSENT

Written informed consent was obtained from the patient for their anonymized information to be published in this article.

## Data Availability

The data that support the findings of this study are available from the corresponding author upon reasonable request.
